# Cross-species hybrid vesicles enable synergistic immunochemotherapy through enhanced targeted drug delivery and immune activation

**DOI:** 10.1186/s12951-026-04699-2

**Published:** 2026-06-19

**Authors:** Zhou Fang, Saichao Wei, Shaoqian Wang, Jiaqi Xu, Min Zhang, Mingyu Yin, Jun Wang, Kehai Liu

**Affiliations:** 1 Department of Biopharmaceutical Science, Shanghai Ocean University, Hucheng Ring Road, Shanghai, China; 2 School of Medicine, The International Peace Maternal and Child Health Hospital, Shanghai Jiao Tong University, Shanghai, China; 3 Marine Biomedical Science and Technology Innovation Platform of Lin- gang Special Area, Shanghai, China

**Keywords:** Hybrid vesicles, Targeting, DC maturation, Immunochemotherapy, Combination therapy

## Abstract

To overcome the critical challenges of insufficient drug targeting and restricted dendritic cell (DC) activation in chemo-immunotherapy, this study developed an innovative cross-species hybrid vesicle delivery system. By fusing extracellular vesicles derived from 4T1 tumor cells (TEV) with kiwifruit-derived extracellular vesicles (KEV), we constructed a homologous targeting hybrid vesicle carrier (TKEV). This platform was co-loaded with chemotherapeutic agent doxorubicin (DOX) and Bcl-2 siRNA (siBcl2) to form a combination therapeutic system (TKDS). Key findings demonstrate: (1) TKDS achieves precise tumor-targeted delivery, significantly enhancing tumor cell apoptosis through combined chemo-gene therapy; (2) The KEV component effectively stimulated DC maturation, enhanced antigen presentation, and increased CD8^+^ T cell infiltration in tumors; (3) In a 4T1 murine breast cancer models, TKDS significantly enhanced antitumor efficacy through synergistic immunochemotherapy. To our knowledge, this work provides the first evidence that a cross-species vesicle fusion strategy can simultaneously enhance drug targeting and immune activation, offering a versatile and promising platform for developing highly effective and safer immunochemotherapy regimens.

**Graphical Abstract:**

**Figure:**
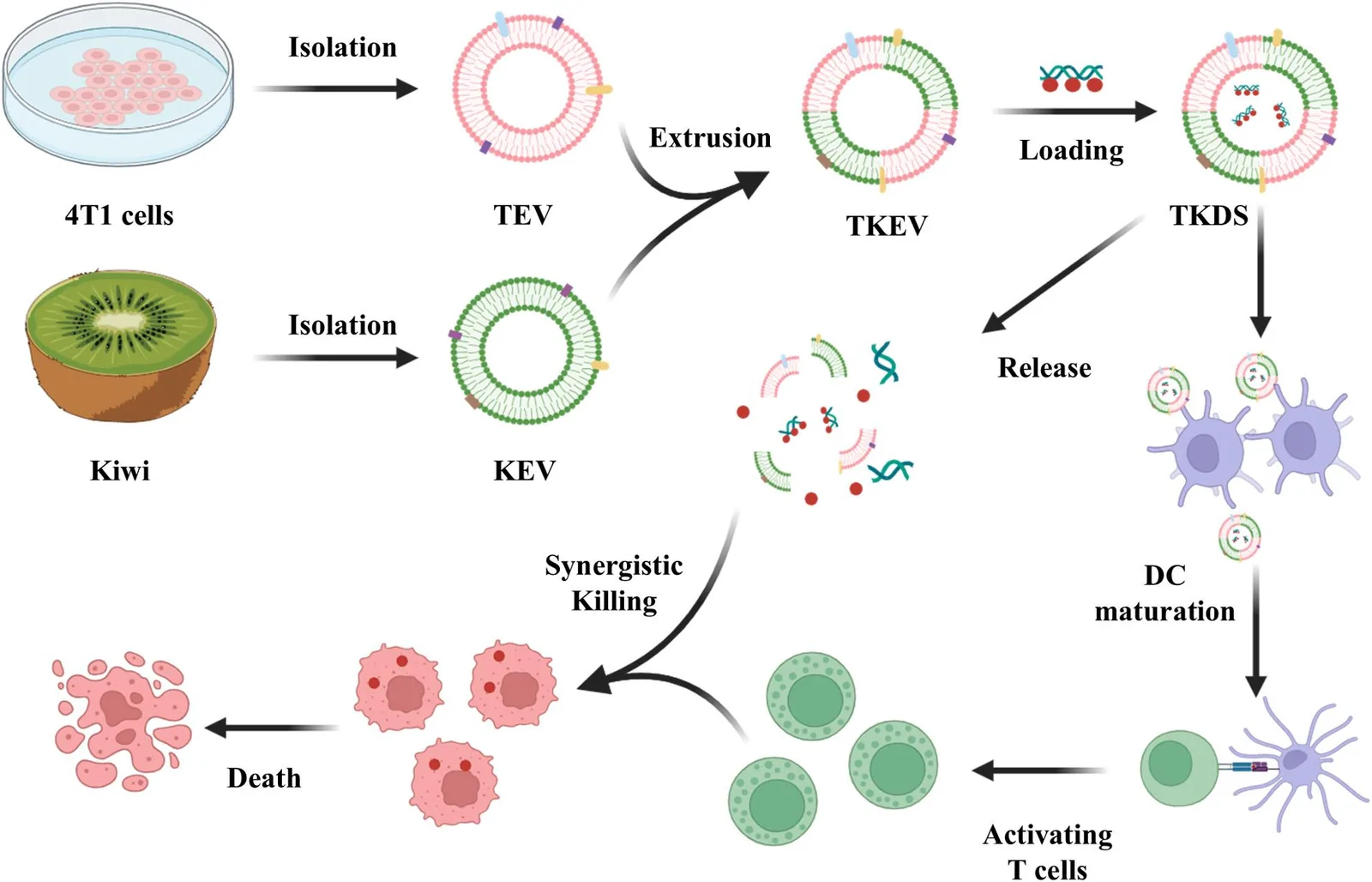

**Supplementary Information:**

The online version contains supplementary material available at https://doi.org/10.1186/s12951-026-04699-2.

## Introduction

Breast cancer, the most prevalent malignancy among women worldwide [1], presents formidable therapeutic challenges due to its high tumor heterogeneity and the development of drug resistance [2]. Although anthracycline-based chemotherapy remains the cornerstone clinical regimen, its efficacy is substantially limited by non-specific biodistribution (which causes systemic toxicity) and insufficient immune activation [3, 4]. Immunochemotherapy, which combines chemotherapy-induced immunogenic cell death (ICD) with immune checkpoint modulation, has emerged as a promising approach to address these issues [5–7]. However, two major obstacles hinder its clinical success: (1) poor tumor-specific targeting leads to suboptimal ICD and restricted release of tumor-associated antigens (TAAs), and (2) inadequate activation of dendritic cell (DC) restricts antigen presentation and subsequent cytotoxic T lymphocyte (CTL) responses. Therefore, developing strategies that enable precise delivery of chemotherapeutic agents while potentiating DC-mediated antigen presentation is critical for advancing immunochemotherapy and represents a key research direction in breast cancer treatment.

Recent advancements in nanocarrier technology have significantly improved drug delivery, with liposomes and polymeric micelles achieving clinical translation [8]. However, such synthetic nanocarriers often suffer from inherent drawbacks, including limited biocompatibility, unpredictable immunogenicity, and an inadequate capacity for sophisticated immune modulation [9]. In contrast, extracellular vesicles (EV), natural nanoscale carriers, have gained attention for their innate biocompatibility and targeting specificity [10, 11]. Tumor-derived EV (TDEV) exploit integrin-mediated homing effects for homologous tumor targeting, while plant-derived EV (PDEV) provide distinct advantages such as immunostimulatory components and scalable production [12–14]. Nevertheless, each single-source EV type exhibits functional shortcoming: TDEV may carry immunosuppressive molecules (e.g., PD-L1, TGF-β), and PDEV lack tumor-specific targeting capability. This functional paradox underscores the promise of hybrid animal–plant EV systems, which combine the targeting precision of TEV with the immunostimulatory and biosafety advantages of PEV through membrane fusion technology, thereby addressing the limitations of both synthetic carriers and single-source EV.

DCs, the most potent antigen-presenting cells, play a pivotal role in determining the magnitude of antitumor immune responses. Studies have demonstrated that chemotherapy-released TAAs must be efficiently captured, processed, and presented by DCs to initiate CTL activation [15, 16]. However, immunosuppressive factors (e.g., IL-10, TGF-β) within the tumor microenvironment (TME) can induce DC dysfunction, ultimately leading to immune tolerance [17]. Notably, PEV have shown remarkable potential in activating DCs. For instance, extracellular vesicle-like nanoparticles from *Sargassum fusiforme* (SF-NPs) promote DC maturation [18], while *Petasites japonicus*-derived EVs enhance DC activation [19]. Additionally, garlic-derived nanoparticles have been found to potentiate γδ T cell responses [20]. In our previous work, kiwifruit-derived EVs (KEV) were identified as potent inducers of DC maturation, providing a theoretical foundation for designing hybrid EV systems with dual delivery and immunomodulatory capabilities [21].

DOX, a classical anthracycline chemotherapeutic, intercalates into DNA and inhibits topoisomerase II, remaining a cornerstone in the systemic management of advanced breast cancer [22]. Although neoadjuvant chemotherapy regimens containing DOX (e.g., TAC, AC) improve the pathological complete remission (pCR) rates, the 5-year overall survival (OS) remains unsatisfactory (below 40%) [23, 24]. This therapeutic plateau is largely attributed to tumor cell evasion of apoptosis, frequently mediated by overexpression of the anti-apoptotic protein Bcl-2. Elevated Bcl-2 expression confers resistance to apoptotic signals through multiple mechanisms, including regulation of mitochondrial membrane permeability and suppression of pro-apoptotic protein activation [25, 26]. Consequently, targeting the Bcl2-mediated anti-apoptotic pathway represents a promising therapeutic avenue for overcoming DOX resistance.

**Fig. 1:**
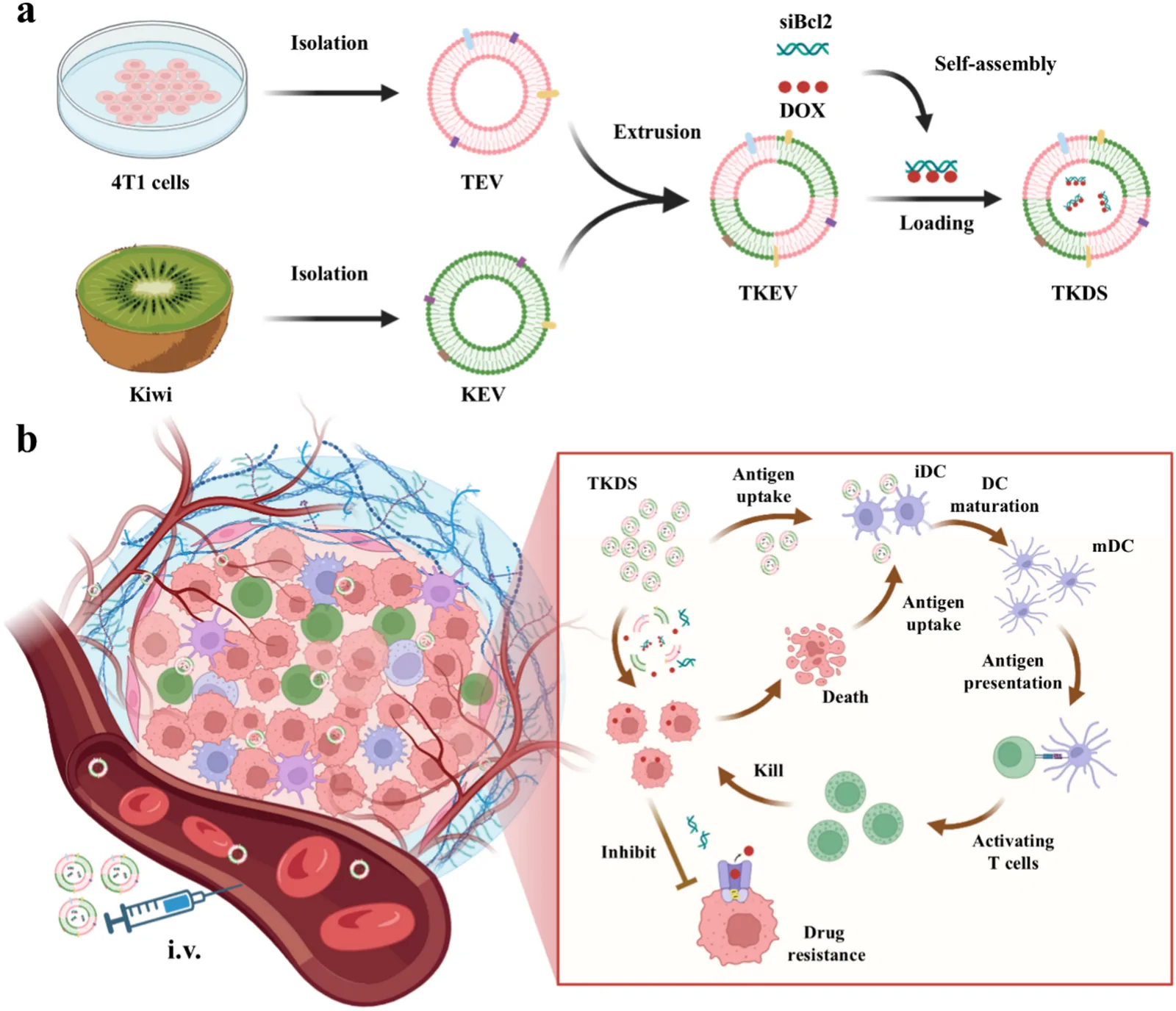
Schematic illustration of TKDS for tumor inhibition based on hybrid vesicle-based co-delivery of DOX and siBcl2. (**a**) Preparation of TKDS by co-extrusion of TEV and KEV followed by loading with DOX/siBcl2. **(b**) Mechanism of action of TKDS in immunochemotherapy for tumor suppression. (Created with BioRender.com)

Herein, we propose a novel “biohybrid immunochemotherapy” strategy based on an engineered a hybrid vesicle (TKEV), constructed via membrane fusion of 4T1 cell-derived TEV and kiwifruit-derived KEV, and co-loaded with DOX and siBcl2 to form a multifunctional platform (TKDS) (Fig. 1a, b). Systematic investigations revealed three key findings: (1) Hybrid EV retained parental membrane proteins and exhibited 3.6-fold increase in tumor accumulation compared to free DS; (2) KEV components potently promoted DC maturation, increasing the proportions of CD80^+^ and CD86^+^ cells by 43% and 67%, respectively; (3) Co-delivery of DOX and siBcl2 synergistically induced apoptosis, resulting in a 136% increase in the apoptotic rate. To our knowledge, this is the first study to demonstrate the feasibility of animal-plant EV fusion for cancer therapy, which concurrently overcomes drug resistance and immunosuppression, leading to significant tumor growth inhibition in murine breast cancer models. These findings establish a blueprint for developing multifunctional EV-based systems that transcend biological boundaries, offering new avenues for personalized cancer therapeutics.

## Results and discussion

### Preparation and characterization of TKEV

**Fig. 2:**
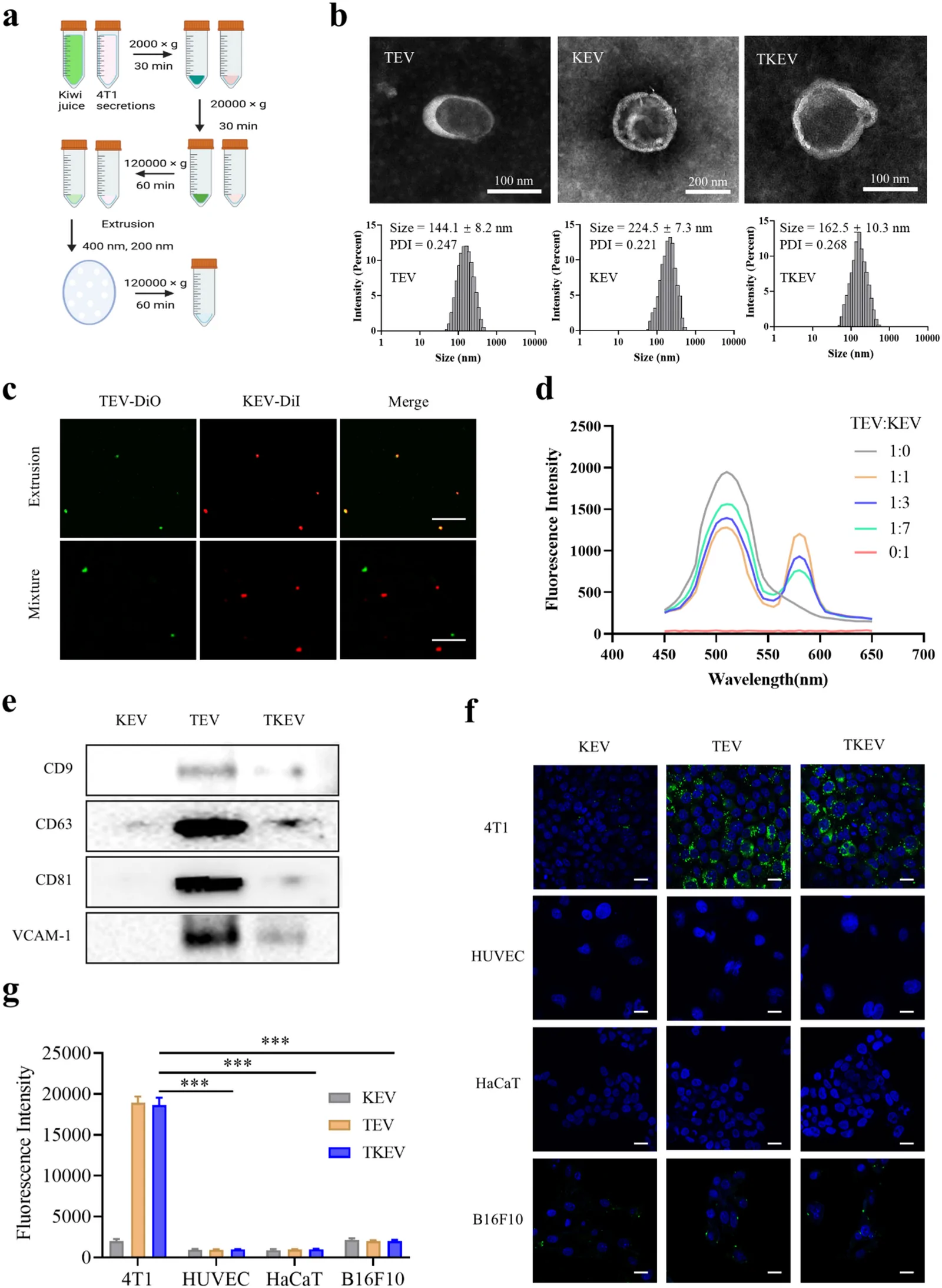
Preparation and characterization of TKEV. (**a**) Schematic illustration of the preparation of TEV, KEV and TKEV. (**b**) TEM images and size distributions of TEV, KEV and TKEV. (**c**) Representative CLSM images of physical mixture (TEV and KEV) and fused TKEV (prepared by co-extrusion) (TEV: KEV = 1 : 3). Green: TEV labeled with DiO; red: KEV labeled with DiI. Scale bar = 20 μm. (**d**) FRET spectra of fused TEV/KEV hybrids with increasing amounts of KEV. (**e)** Western blot analysis of specific antigen expression in KEV, TEV and TKEV. **(f, g**) cellular uptake of different formulations in 4T1, HUVEC, HaCaT, and B16F10 cells as evaluated by CLSM (scale bar = 20 μm) and flow cytometry (*n* = 5; ***p < 0.001.)

TKEV was prepared through co-extrusion of TEV and KEV (Fig. 2a). TEV and KEV were first isolated separately via differential centrifugation according to an established protocol. The two vesicles were mixed at varying ratios and then extruded through 0.4 μm and 0.2 μm polycarbonate membranes to obtain the hybrid TKEV. To mitigate the potential risk of immunosuppressive molecules carried by TEV, we subjected TEV to multiple freeze-thaw cycles to release its contents. The results showed that multiple freeze-thaw cycles effectively eliminated TGF-β from TEV (Figure S1). The morphology and size distribution of the resulting hybrid vesicles were analyzed using transmission electron microscopy (TEM) and dynamic light scattering (DLS). TEM imaging showed that both TEV and KEV exhibited the typical cup-shaped morphology of extracellular vesicles, while TKEV maintained their spherical vesicle structure. Consistent with the TEM observations, DLS measurements indicated that the average hydrodynamic diameters of TEV and KEV were approximately 144.1 nm and 224.5 nm respectively, whereas TKEV showed an intermediate size of around 162.5 nm (Fig. 2b). All formulations exhibited a PDI of less than 0.3, indicating a uniform size distribution and no significant aggregation.

To confirm the fusion of TEV and KEV, the vesicles were labeled with DiO (green membrane dye) and DiI (red membrane dye), respectively, and visualized using confocal laser scanning microscopy (CLSM). As shown in Fig. 2c, simply mixed TEV and KEV exhibited dispersed fluorescence signals, whereas TKEV prepared by sequential co-extrusion showed complete overlap of green and red fluorescence, indicating successful membrane fusion. The fusion process was further evaluated using Förster resonance energy transfer (FRET) analysis. Figure 2d illustrates that increasing the amount of KEV resulted in enhanced donor (DiO) recovery and concomitant acceptor (DiI) quenching, confirming efficient fusion between TEV and KEV and a corresponding reduction in FRET interaction. To optimize the optimal fusion ratio, the targeting performance and serum stability of TKEV were simultaneously examined. The results indicated that a higher KEV content in the hybrid vesicles was associated with reduced uptake efficiency by 4T1 cells but improved serum stability (Figures S2 and S3). Based on the homologous targeting performance and serum stability, a weight ratio of TEV to KEV of 1:3 was identified as the optimal formulation. Furthermore, DiO and DiD were used to label TEV and KEV, respectively, to quantify the composition of TEV and KEV in TKEV. The results showed that the final composition of TEV and KEV was generally consistent with the feed ratio (Figures S5).

Breast cancer cell 4T1-derived vesicles, exhibit tumor-specific targeting capabilities owing to the retention of membrane components from their parent cells, such as the vascular cell adhesion molecule VCAM-1, which is implicated in cellular adhesion and migration. Western blot analysis confirmed that the hybrid TKEV preserved characteristic proteins of its TEV component, including CD9, CD63, CD81, and VCAM-1 (Fig. 2e, S4). Given previous reports highlighting the exceptional stability of KEV [21], we further investigated the stability profile of TKEV. Dynamic monitoring of particle size revealed a significant increase in the diameter of TEV—from 144.1 ± 8.2 nm to 236.5 ± 21.4 nm—after 7 days of storage in buffer (*p* < 0.001). In contrast, KEV and TKEV maintained high structural stability, with no significant change in size (223.3 ± 7.3 nm vs. 228.8 ± 6.9 nm, *p* > 0.05; 162.5 ± 10.3 nm vs. 168.9 ± 12.7 nm, *p* > 0.05) (Figure S6). These results suggests that the integration of KEV enhances the structural integrity of the hybrid vesicles. This improved stability is likely attributed to the presence of phosphatidylcholine (PC) and phosphatidylethanolamine (PE) lipids derived from KEV [21].

To investigate the targeting selectivity of TKEV, we compared the cellular uptake efficiency of different vesicles in 4T1 (homologous tumor cells), HUVECs (human umbilical vein endothelial cells), HaCaT (human immortalized keratinocytes), and B16F10 (murine cutaneous melanoma) cells. As shown in Fig. 2f and g, both TEV and TKEV were efficiently internalized by 4T1 cells, whereas significantly less internalization was observed in HUVECs, HaCaT, and B16F10 cells. These findings indicate that the hybrid TKEV retain the ability to selectively target their parental 4T1 cells while minimizing uptake by off-target cell types. This targeting specificity may originate from (1) the conserved presence of VCAM-1 on TKEV, which facilitates homologous targeting, and (2) charge shielding contributed by KEV components that reduces nonspecific cellular uptake. This selective targeting capability supports enhanced tumor accumulation together with reduced off-target toxicity, making TKEV a promising platform for precision drug delivery.

### Drug loading and in vitro release study

**Fig. 3:**
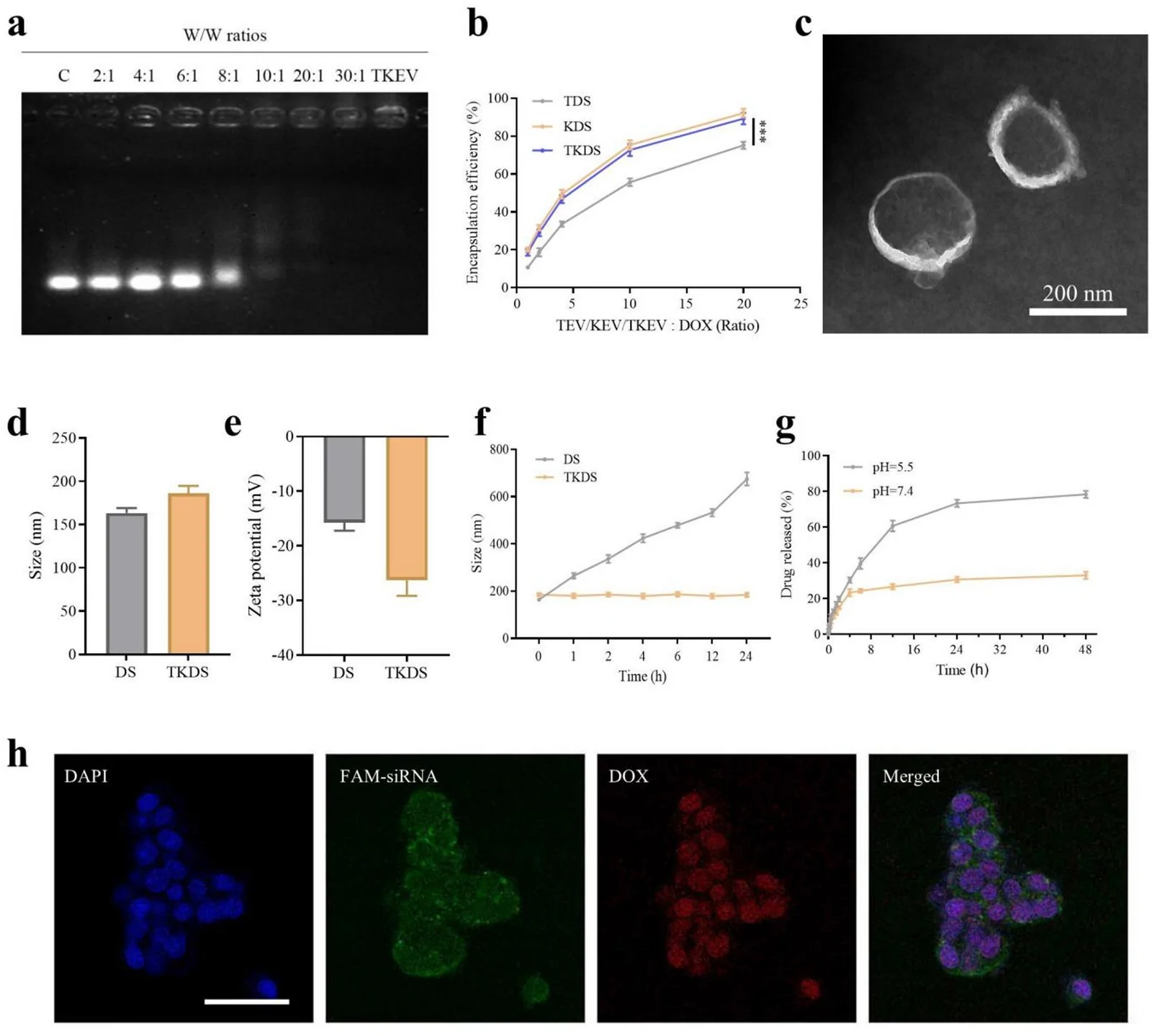
Drug loading and release profiles of TKDS. (**a**) Complexation efficiency of siBcl2 with DOX. (**b**) Encapsulation efficiency of DOX in TEV, KEV and TKEV at different ratio (*n* = 5). (**c)** TEM image of TKDS. (**d**) Size distribution of DS and TKDS (*n* = 5). (**e**) Zeta potentials of DS and TKDS (*n* = 5). (**f**) Stability of TKDS in 10% fetal bovine serum (FBS) over time, as measured by size change (*n* = 5). (**g**) In vitro release profiles of DOX from TKDS at pH 5.5 and pH 7.4 (*n* = 5). (**h**) Representative CLSM images of 4T1 cells after incubated with TKDS. Scale bar = 50 μm

DOX is a widely used clinical chemotherapeutic, and overexpression of the anti-apoptotic Bcl-2 gene is frequently implicated in tumor chemoresistance. Co-delivery of DOX and siBcl2 has previously been demonstrated to significantly enhance antitumor efficacy [27]. In this study, DOX and siBcl2 were condensed via electrostatic interactions to form nanocomplexes (DS), with a diameter of 163.2 nm and a zeta potential of -15.8 mV. These DS complexes were subsequently loaded into TKEV using co-extrusion, yielding TKDS particles with a diameter of 186 nm and a zeta potential of -26.3 mV (Fig. 3d and e). The condensation of siBcl2 by DOX was assessed using agarose gel electrophoresis. Results indicated that increasing the DOX feeding ratio led to progressive complex formation and siRNA retardation. At a DOX/siRNA weight ratio of 10:1, siBcl2 was almost entirely retained in the gel spiking wells, and this ratio was selected for all subsequent experiments (Fig. 3a). We further compared the drug loading capacity across different vesicle carriers (TDS, KDS, and TKDS). The encapsulation efficiency of TKDS increased linearly from 18.6% to 89.2% with higher DS feeding ratios, which was significantly greater than that of TDS (maximum 75.2%, *p* < 0.001) and comparable to that of KDS (92.2%, *p* > 0.05) (Fig. 3b). This suggests that membrane components of KEV, such as phosphatidylcholine (PC) and phosphatidylethanolamine (PE), which contribute to high lipid rigidity, play a critical role in enhancing drug encapsulation [28]. As shown in Fig. 3c, TKDS retained intact vesicular morphology after dual-drug loading, supporting its potential for efficient delivery.

Serum stability assays indicated that TKDS maintained excellent colloidal stability, with less than 5% variation in particle size over 24 h in DMEM supplemented with 10% fetal bovine serum. In contrast, free DS complexes aggregated rapidly due to protein adsorption, exhibiting a greater than 200% increase in particle size under the same conditions (Fig. 3f). These results confirm that encapsulating DS within TKEV effectively shields the cargo from destabilizing factors in biological environments. Drug release kinetics analysis revealed a dual pH-responsive behavior of TKDS: the cumulative release over 24 h was only 31 ± 1.5% at physiological pH (7.4), but increased markedly to 78.3 ± 2.3% under acidic lysosomal conditions (pH 5.5) (Fig. 3g). This smart release behavior can be attributed to: (1) Protonation-induced lipid phase transition within the TKEV membrane under acidic conditions, enhancing membrane permeability, and (2) In an acidic environment, protonation of the tertiary amine group of DOX facilitates its dissociation and release from the TKEV vesicle. Confocal imaging further demonstrated that TKDS-mediated delivery resulted in precise subcellular compartmentalization—DOX was localized primarily in the nucleus, while siBcl2 was distributed in the cytoplasm (Fig. 3h). This spatiotemporally controlled release pattern enables synchronized induction of DNA damage (by DOX) and suppression of anti-apoptotic signaling (by siBcl2), offering a novel synergistic strategy to overcome multidrug resistance.

### Intracellular trafficking of TKDS

**Fig. 4:**
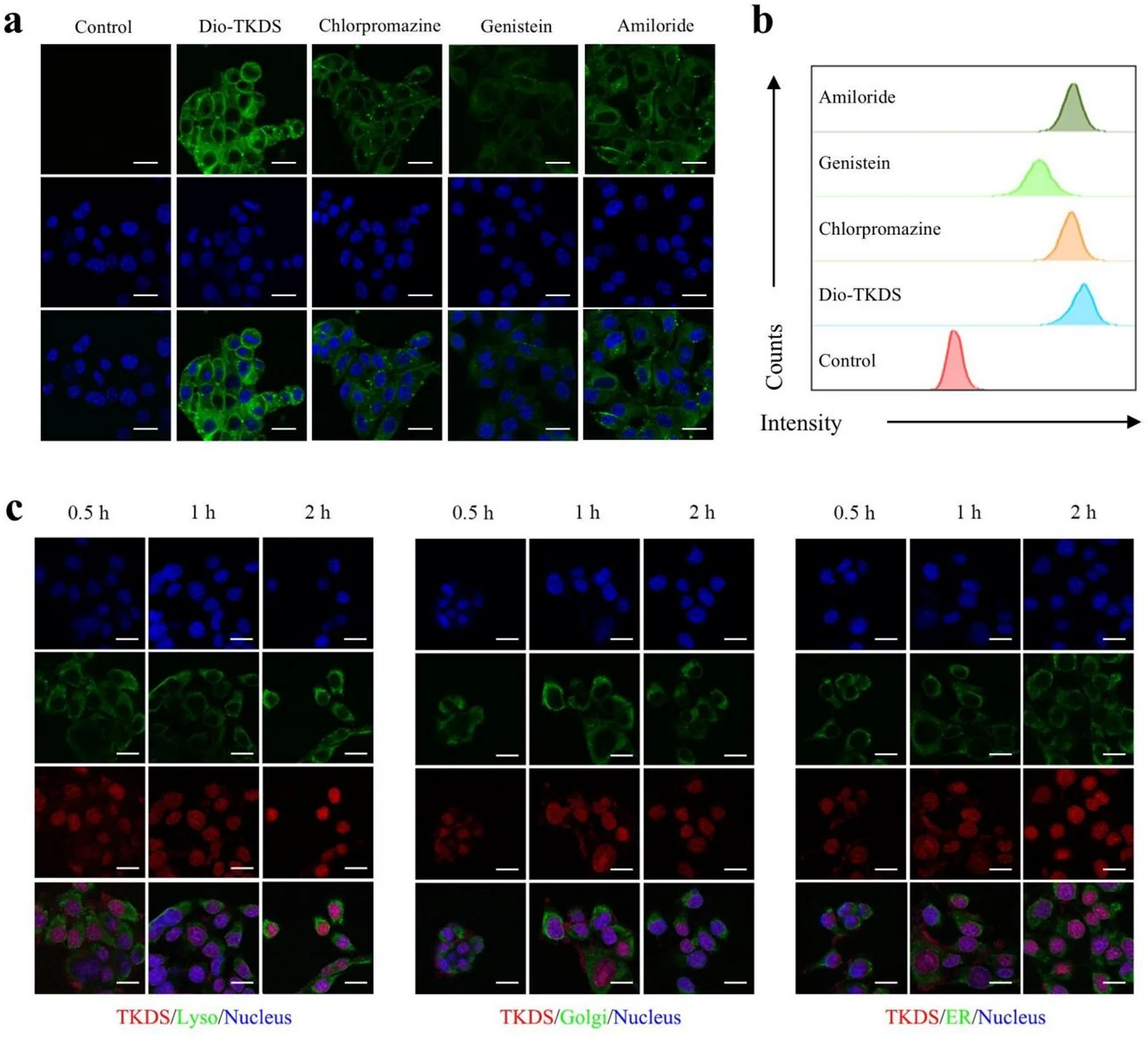
Intracellular trafficking of TKDS. (**a**) Representative CLSM images of TKDS uptake in 4T1 cells after treatment with different endocytosis inhibitors. Scale bar = 10 μm. (**b**) Quantitative analysis of TKDS internalization pathways inhibited by various endocytosis inhibitors (*n* = 5). (**c**) Representative CLSM images of 4T1 cells incubated with TKDS for 0.5 h, 1 h, and 2 h. Nuclei were stained with DAPI (blue), and lysosomes, Golgi apparatus and endoplasmic reticulum were labeled with anti-LAMP1, anti-Golgin-97, and anti-KDEL antibodies, respectively. Scale bar = 10 μm

The intracellular trafficking of TKDS plays a crucial role in mediating the synergistic effects of DOX and siBcl2. To elucidate this process, we systematically investigated the cellular internalization pathways of TKDS in 4T1 cells using pharmacological inhibitors of specific endocytic mechanisms. Endocytosis inhibition assays revealed that caveolae-mediated endocytosis was the predominant uptake route, accounting for 84.1% of internalization (inhibited by genistein). This was significantly higher than the contributions of clathrin-dependent endocytosis (35.3%, inhibited by chlorpromazine, CPZ) and macropinocytosis (40.4%, inhibited by amiloride) (Fig. 4a and b). These results indicate that TKDS is primarily internalized via caveolae-mediated endocytosis, with clathrin-dependent uptake and macropinocytosis playing secondary roles in the overall cellular entry process.

To elucidate the subcellular trafficking of TKDS, lysosomes, Golgi apparatus, and endoplasmic reticulum (ER) were labeled using fluorescent antibodies against LAMP1, Golgin-97, and KDEL, respectively. As shown in Fig. 4c and S7, partial co-localization of DOX (red fluorescence) with lysosomes (green fluorescence) was observed after 0.5 and 1 h of incubation with 4T1 cells. Over time, the lysosomal colocalization decreased while nuclear accumulation (blue fluorescence) of DOX increased, indicating that TKDS facilitates lysosomal escape and promotes efficient nuclear delivery of DOX. Furthermore, a strong TKDS signal was detected in the Golgi apparatus within 0.5 h, which gradually shifted toward the ER and nucleus at later time points. These results suggest that TKDS mediates drug delivery to the nucleus via both lysosomal escape and the Golgi–ER pathway, enhancing overall drug efficacy. Additionally, the Golgi-ER route may promote intercellular drug transport within solid tumors, thereby improving drug penetration [29].

### Immune activation of TKDS

**Fig. 5:**
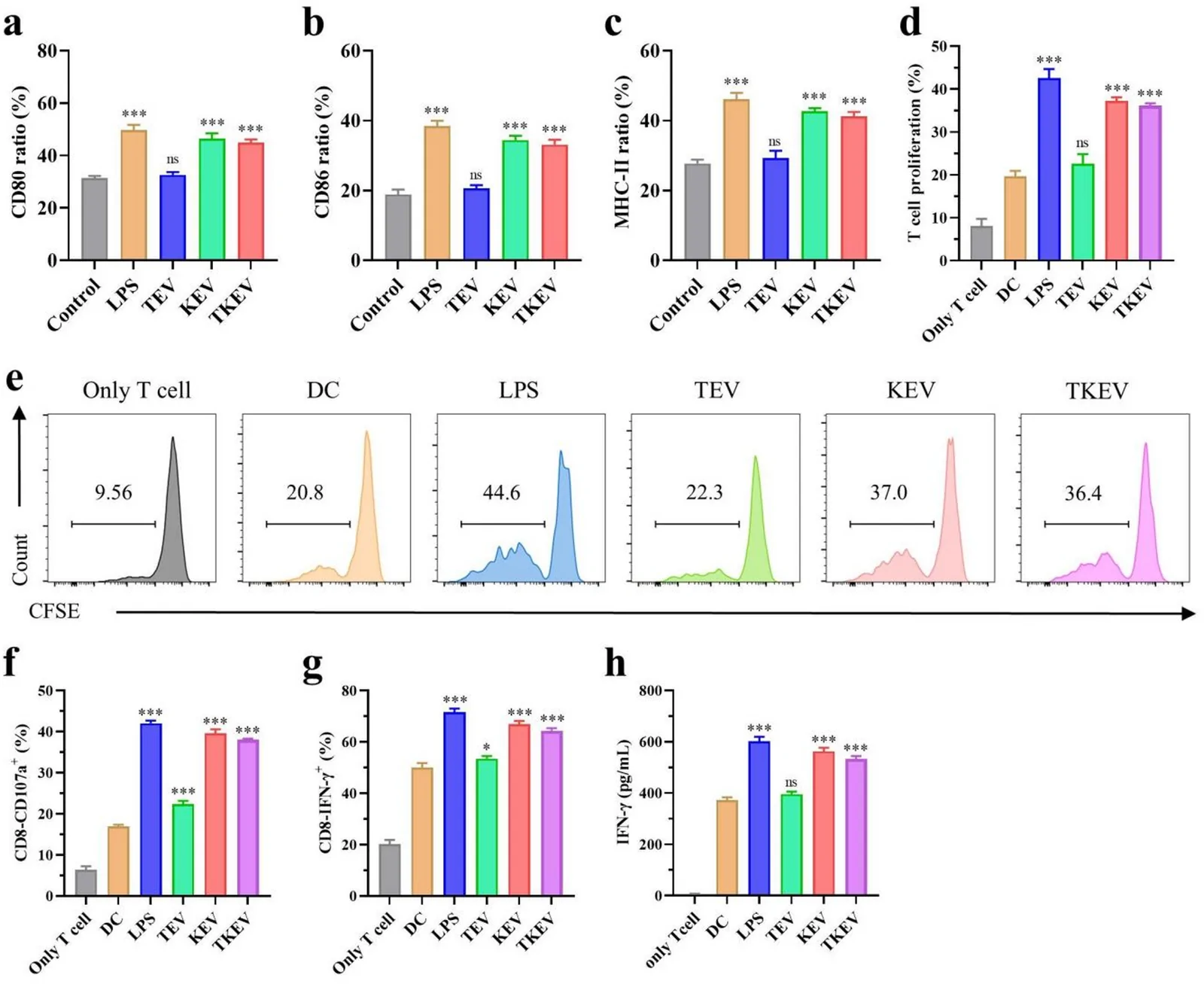
Effects of different treatments on dendritic cell (DC) and T cell activation. (**a-c**) Quantitative analysis of mature DC markers (CD80, CD86 and MHC-II) following 24 h treatment with PBS, LPS, TEV, KEV and TKEV. **(d, e)** Representation flow cytometry plots and quantitative analysis of CD8^+^ T cell proliferation. (**f, g**) Percentages of CD107a^+^ and IFN-γ^+^ cells among CD8^+^ T cells. (**h)** Cytokine expression levels in culture supernatants were quantified by ELISA. Data are presented as mean ± SD (*n* = 5). ***p < 0.001

To evaluate the immune activation effect of TKEV, we examined its ability to induce DC maturation, T cell proliferation and T cell activation in vitro. Flow cytometry analysis revealed that treatment with TKEV significantly upregulated the expression of the costimulatory molecules CD80 and CD86, as well as MHC-II, on DCs, to levels 1.4-fold, 1.8-fold, and 1.5-fold higher than those in the control group, respectively (*p* < 0.001), reaching 88.6% of the effect observed with LPS positive control (Fig. 5a, b and c, and S8). We further investigated the impact of TKEV-treated DCs on T cell responses using a mixed lymphocyte reaction (MLR). Results showed that DCs pretreated with TKEV enhanced CD8⁺ T cell proliferation by 1.8-fold compared to the untreated group (*p* < 0.001), whereas untreated DCs elicited minimal T cell proliferation (Fig. 5d, e). The cytolytic activity of CD8⁺ T cells were assessed by measuring the expression of CD107a (a degranulation marker) and IFN-γ. Flow cytometry indicated that both KEV and TKEV treatment promoted CD107a and IFN-γ expression in CD8⁺ T cells at levels comparable to those induced by LPS (Fig. 5f, g and S9). Consistent results were obtained using ELISA (Fig. 5h).

The underlying mechanism by which TKEV induces DC maturation and subsequent T-cell activation warrants further discussion. Accumulating evidence has demonstrated that plant-derived extracellular vesicles can promote DC phenotypic maturation and functional activation via the mitogen-activated protein kinase (MAPK) and nuclear factor-κB (NF-κB) pathways. For instance, EVs derived from *Sargassum fusiforme* have been shown to significantly upregulate the expression of co-stimulatory molecules (CD80, CD86, and MHC-II) on DCs in a manner dependent on MAPK and NF-κB activation and phosphorylation. Moreover, DCs primed by plant-derived EVs effectively induce the proliferation and differentiation of naïve T cells into cytotoxic CD8⁺ T lymphocytes, accompanied by robust secretion of IFN-γ and IL-2 [18]. This mechanistic framework has been consistently documented across multiple studies of plant-derived vesicles [19]. In the present work, TKEV—as a hybrid vesicle incorporating kiwifruit-derived membrane components—exhibited immunostimulatory properties closely resembling those of plant-derived EVs, suggesting that its biological activity likely involves engagement of analogous signaling cascades. Collectively, these findings establish TKEV as a potent inducer of DC maturation, thereby facilitating T-cell proliferation and activation, and ultimately driving robust antitumor immune responses.

### Induction of tumor cell death of TKDS

**Fig. 6:**
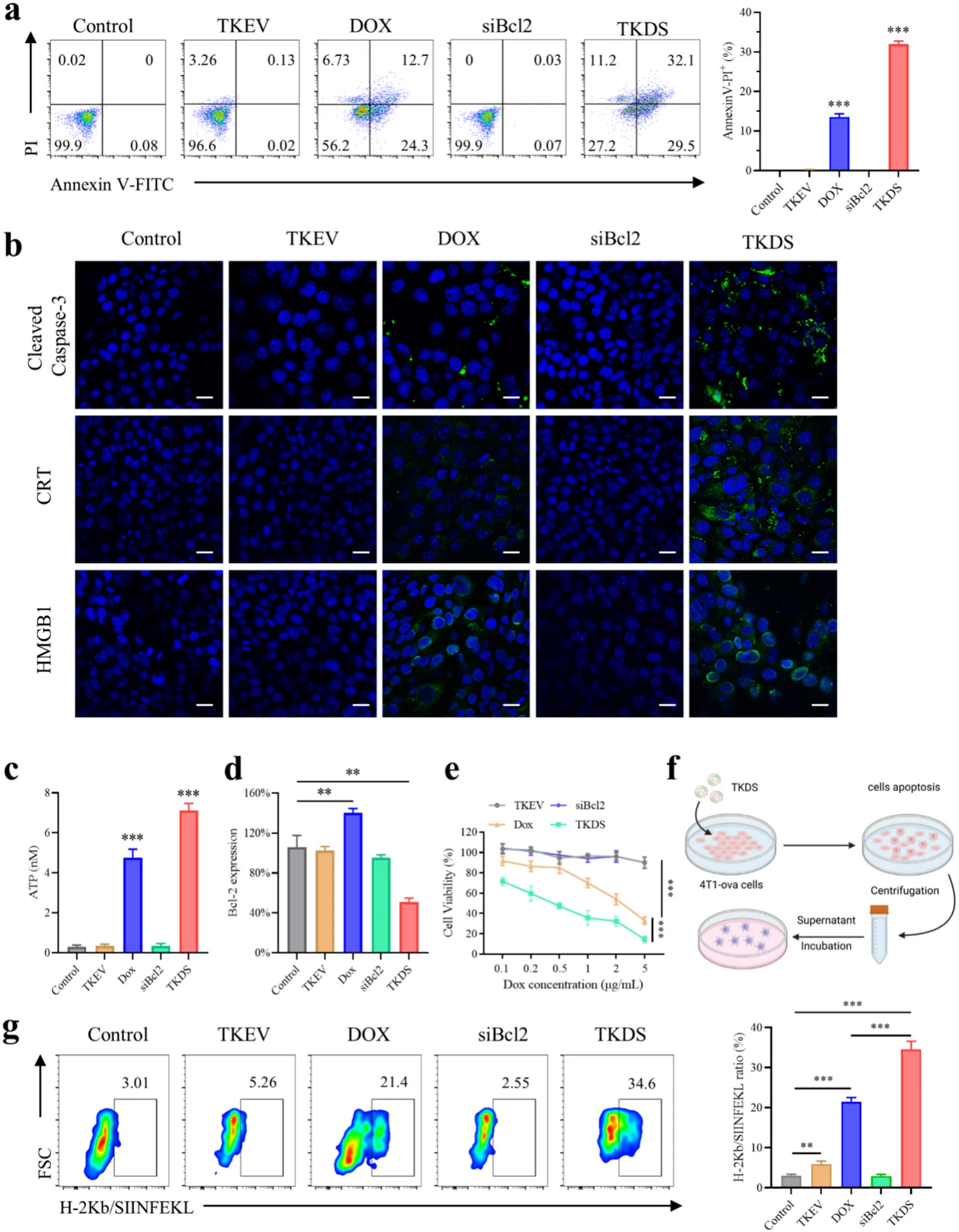
In vitro antitumor efficiency of TKDS. (**a**) Representative flow cytometry plots and quantitative analysis of apoptosis in 4T1 cells after different treatments. (**b**) Representative immunofluorescence images of cleaved caspase-3, CRT, and HMGB1 in 4T1 cells treated with the indicated formulations. Scale bar = 20 μm. (**c**) Extracellular ATP levels released from 4T1 cells after various treatments, measured by chemiluminescent ATP assay kit (*n* = 5). (**d**) Bcl2 mRNA expression levels in 4T1 cells treated with different formulations, as determined by RT-qPCR (*n* = 5). (**e**) Viability of 4T1 cells after incubation with different formulations for 48 h (*n* = 5). (**f**) Schematic illustration of enhanced antigen presentation mediated by TKDS treatment. (**g**) Representative flow cytometry plots and quantitative analysis of antigen presentation levels

To investigate the antitumor efficacy of TKDS in vitro, we examined its effects on apoptosis and Bcl-2 expression in 4T1 cells. Flow cytometry analysis revealed that TKDS treatment induced apoptosis in 32.1% of cells, a rate twice as high as that achieved with free DOX. In contrast, siBcl2 alone showed negligible inhibition of cancer cell growth (Fig. 6a). Additionally, we stained cells from different treatment groups using a cleaved caspase-3 antibody. The results showed positive fluorescence in the DOX and TKDS treatment groups, with the TKDS group exhibiting the strongest fluorescence, consistent with the apoptosis results detected by flow cytometry. To validate chemotherapy-induced immunogenic cell death of tumor cells, we measured the major hallmarks of immunogenic cell death. Calreticulin (CRT) and HMGB1 were labeled by immunofluorescence staining, and ATP release was determined using a chemiluminescent ATP assay kit. Compared with the control group, both DOX and TKDS treatment groups significantly induced the translocation of CRT to the cell membrane surface, the release of HMGB1, and the increase of ATP secretion levels (*p* < 0.001), while the TKEV and siBcl2 groups showed no significant changes. This result confirms that TKDS can effectively induce ICD in tumor cells (Fig. 6b and c). Further analysis indicated that DOX treatment led to an increase in Bcl-2 expression, likely reflecting a compensatory resistance mechanism. While siBcl2 alone did not significantly affect Bcl-2 levels, TKDS treatment resulted in a pronounced reduction of Bcl-2 mRNA expression, with a knockdown efficiency reaching 48.9% (Fig. 6d). We next assessed the effect of TKDS on cell viability. As expected, TKDS significantly inhibited the growth of 4T1 cells through combined DOX delivery and Bcl-2 silencing, reducing cell viability by 2.3-fold compared to DOX alone and by 6.1-fold compared to siBcl2 alone. TKEV vesicles without drug loading showed minimal impact on proliferation (Fig. 6e). To investigate whether TKDS-treated 4T1 cells could enhance antigen presentation by DCs, DCs were incubated with the supernatant from TKDS-treated 4T1 cells (Fig. 6f). The results showed that TKDS treatment significantly enhanced the presentation of antigen peptides by DCs (Fig. 6g), indicating that TKDS induces enhanced immunogenic cell death in tumor cells and subsequently activates DCs.

### In vivo anti-tumor ability of TKDS

**Fig. 7:**
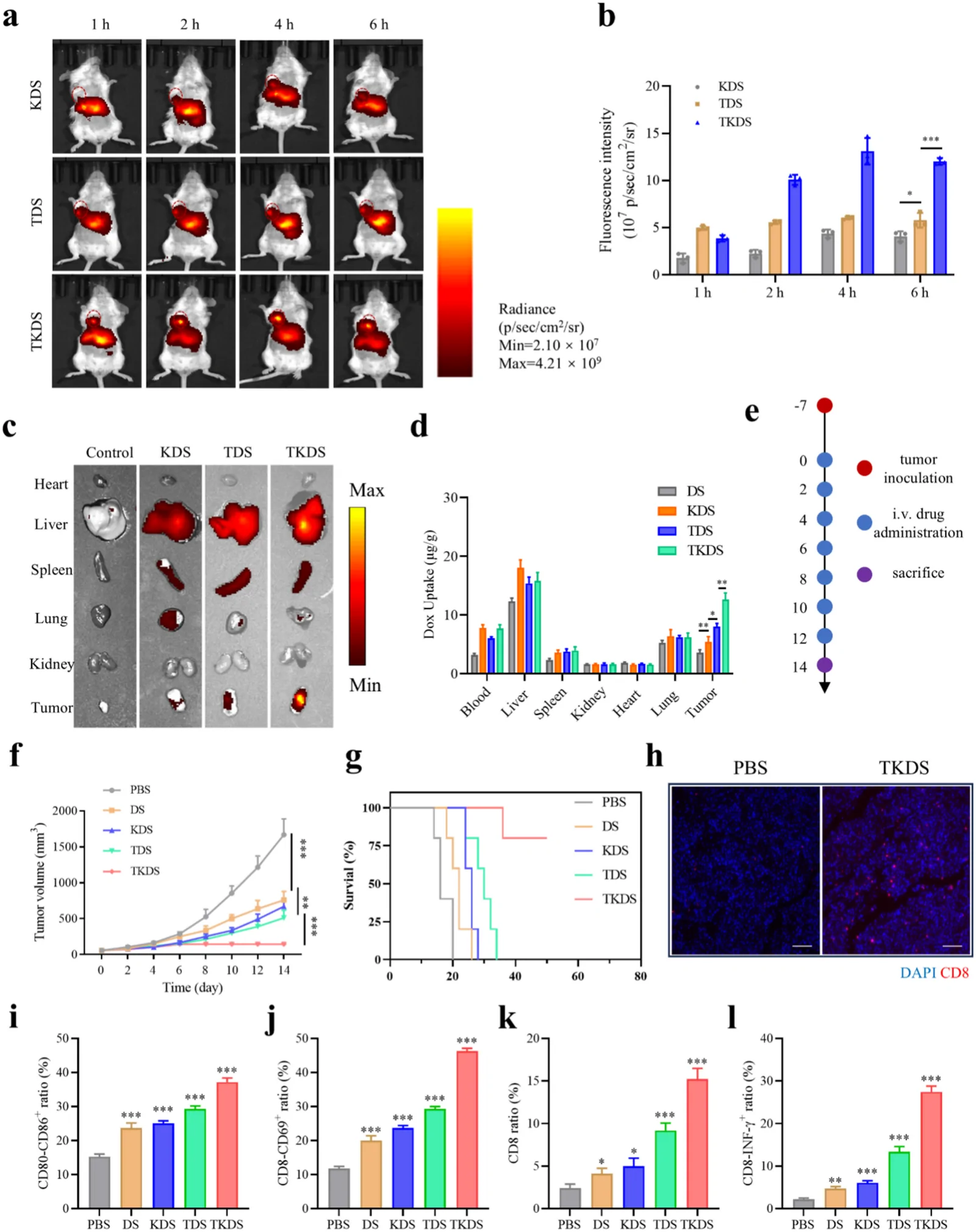
In vivo antitumor efficacy of TKDS. (**a, b**) Representative in vivo fluorescence images and quantitative analysis of tumor-bearing 4T1 mice at 1, 2, 4 and 6 h post intravenous injection of DiR-labeled KDS, TDS, or TKDS. (**c**) Ex vivo fluorescence images of major organs and tumors collected 6 h post-injection. (**d**) DOX concentration in tissues after intravenous administration (*n* = 5). (**e**) Schematic of the experimental timeline. BALB/c mice were subcutaneously inoculated with 1 × 10^6^ 4T1 cells and treated when tumor volume reached approximately 50 mm^3^. (**f**) Tumor growth curves following treatment with different formulations (*n* = 5). (**g**) Survival analysis of tumor-bearing mice after different treatments. (*n* = 5). (**h**) Representative immunofluorescence images of CD8^+^ T cells infiltrating in tumor tissues. Scale bar: 50 μm. (**i**) proportion of mature DCs in tumor-draining lymph nodes (TDLN). (**j**) Percentage of activated (CD69) CD8^+^ T cells in TDLN. (**k**) Infiltration of CD8^+^ T cells in the tumor microenvironment. (**l**) Proportion of IFN-γ^+^ CD8^+^ T cells among tumor-infiltrating lymphocytes. Data are presented as mean ± SD; *p < 0.05; **p < 0.01; ***p < 0.001

To investigate the specific targeting capability of TKEV in vivo, we assessed the biodistribution of DiR-labeled TKDS in a 4T1 subcutaneous xenograft model. Our results showed that TKEV reduced nonspecific accumulation in major organs while enhancing tumor-specific targeting. As shown in Fig. 7a and b, both TKDS and TDS exhibited significant tumor accumulation as early as 1 h post-injection. The signal intensity continued to increase over time, plateauing at 4 h, with a noticeably stronger signal observed for TKDS compared to TDS. Ex vivo tissue imaging and tumor tissue fluorescence imaging data both revealed the highest tumor accumulation of TKDS (Figs. 7c and S10). To investigate the reason for the inferior tumor accumulation of TDS compared to TKDS, we evaluated the stability of KDS, TDS, and TKDS in PBS containing 10% serum. The results showed that after 4 h of incubation in serum-containing PBS, the particle size of TDS increased significantly, whereas KDS and TKDS remained almost unchanged (Figure S11). Therefore, the suboptimal tumor accumulation observed in the TDS group can be attributed to its reduced systemic stability, which facilitates rapid clearance and compromises tumor delivery.

We also measured the plasma concentration of DOX in different formulations at 6 h post-injection. At this single time point, the plasma concentration of DOX encapsulated in TKEV was significantly higher than that of free DS, suggesting a potential for prolonged circulation conferred by vesicle encapsulation. Furthermore, the tumor accumulation of TKDS was 3.6-fold, 2.3-fold, and 1.6-fold higher than that of free DS, KDS, and TDS, respectively (Fig. 7d). This enhanced tumor enrichment may be attributed to the superior stability and homologous targeting capacity of TKEV. The relatively higher accumulation of TKDS and KDS in the liver could be associated with their extended blood residence and subsequent hepatic clearance. Together, these findings demonstrate that TKEV encapsulation significantly promotes the targeted accumulation of DOX in tumor tissue.

The antitumor efficacy of TKDS was further assessed in a 4T1 subcutaneous xenograft model (Fig. 7e). As shown in Fig. 7f and g, tumor volume in the TKDS group decreased significantly compared to all other groups, and the survival rate of mice in the TKDS group was 80% at the 50-day observation endpoint, indicating potent inhibition of tumor growth. The limited antitumor effect of KDS was primarily due to its inefficient homologous targeting ability, which was consistent with biodistribution results showing poor tumor accumulation. In contrast, TDS (lacking KEV components) exhibited reduced efficacy, likely attributable to its diminished ability to activate antitumor immunity. Notably, no significant loss in body weight was observed in TKDS-treated mice, indicating a favorable safety profile (Figure S12). Histological examination via H&E staining revealed no obvious pathological changes in major organs, further supporting the minimal systemic toxicity of TKDS (Figure S17). These results demonstrate that TKDS effectively suppresses tumor progression through coordinated delivery of DOX and siBcl2, while exhibiting good biocompatibility in vivo.

### TKDS activates DC maturation to recruit cytotoxic T cells

To assess the capacity of TKDS to activate CD8⁺ T cells, we analyzed DCs and T cell maturation in draining lymph nodes, as well as CD8⁺ T cell infiltration into tumors, using flow cytometry. As shown in Fig. 7i and S13, the proportion of mature DCs was significantly higher in the TKDS group (36.3%) than in the control (16.1%) and TDS (30.2%) groups, indicating that the KEV component enhanced DC maturation. Since DC maturation critically influences T cell activation, we also evaluated the expression of CD69 (an early T cell activation marker) in lymph nodes. The results showed a substantial increase in CD69⁺ T cells in the TKDS (47%) and TDS (30%) groups compared to the control (11.6%) (Fig. 7j and S14). Furthermore, flow cytometric analysis of tumor-infiltrating immune cells revealed the highest percentage of CD8⁺ T cells in the TKDS group (15.2%), compared to 2.12% in PBS, 4.86% in DS, 9.23% in TDS, and 5.98% in KDS groups (Fig. 7k and S15). Immunofluorescence staining confirmed scant T cell infiltration in control tumors, whereas TKDS-treated tumors showed substantial CD8⁺ T cell accumulation (Fig. 7h). To further evaluate the antitumor immune response, we measured IFN-γ expression in tumor-infiltrating CD8⁺ T cells. The TKDS group exhibited the highest IFN-γ expression (28.7%), surpassing all other groups (Fig. 7l and S16). These results demonstrate that TKDS elicits a robust immune response conducive to antitumor immunity.

This study assessed intracellular localization only by single focal‑plane imaging without Z‑stack validation, and the pharmacokinetic data are preliminary based on a single time point; direct in vivo drug‑release visualization was not achieved. Future investigations addressing these aspects would further elucidate the in vivo behavior of TKDS.

## Conclusion

This study develops a novel biohybrid vesicle platform (TKEV) through the fusion of extracellular vesicles derived from 4T1 breast cancer cells (TEV) and kiwifruit (KEV), demonstrating strong synergistic therapeutic effects in breast cancer models. The dual-functional system TKDS, co-loaded with DOX and siBcl2, utilizes TEV-mediated homologous targeting (resulting in a 2.3-fold increase in tumor accumulation) and KEV-induced immune activation via dendritic cell maturation, which led to a 7.2-fold rise in CD8⁺ T cell infiltration. Moreover, TKEV exhibited exceptional colloidal stability, maintaining structural integrity for 7 days, attributable to glycolipid reinforcement from KEV. By integrating biological traits from both animal and plant sources, TKEV introduces a new paradigm in nanomedicine, offering a scalable strategy for next-generation therapies that simultaneously address challenges in drug delivery and immunosuppressive microenvironments across various cancers.

## Methods

### Isolation of TEV and KEV

To obtain extracellular vesicles from 4T1 cells, the cells were cultured in serum-free medium for 48 h. The conditioned medium was then collected and subjected to standard differential centrifugation to obtain TEV. Briefly, the medium was sequentially centrifuged at 300 × g for 10 min, 2000 × g for 10 min, 10, 000 × g for 30 min, 20, 000 × g for 30 min to remove cellular debris and apoptotic bodies. The resulting supernatant was ultracentrifuged at 120, 000 g for 1 h (using a Hitachi CP70-ME ultracentrifuge with a P28s rotor) to pellet TEV. The vesicles were resuspended in PBS and subjected to multiple freeze-thaw cycles to facilitate content release. TEV concentration was determined using a BCA protein assay kit, and aliquots were stored at − 80 ℃. KEV was isolated following a previously established protocol [21].

### Preparation and characterization of TKEV

TKEV prepared by co-extrusion of TEV and KEV. Briefly, TEV and KEV were mixed at a predetermined ratio and extruded multiple times through a liposome extruder (Avestin LF-1, Canada) using 400 nm and 200 nm polycarbonate membrane to facilitate fusion and obtain TKEV.

The successful fusion of TEV and KEV was confirmed by morphological examination using transmission electron microscopy (TEM; Hitachi HT7800, Japan). The particle size distributions of TEV and TKEV were determined via dynamic light scattering (DLS; Malvern Zetasizer Nano ZS, UK). Membrane fusion was further visualized by confocal laser scanning microscopy (CLSM; Leica TCS SP8, Germany) using DiO-labeled TEV (green) and DiI-labeled KEV (red).

Western blotting was performed to characterize specific membrane proteins in TKEV. TEV, KEV, and TKEV samples of equal protein concentration were lysed and denatured in loading buffer. Proteins were separated by SDS-PAGE using the Mini-PROTEAN Tetra system (Bio-Rad, USA) and transferred to PVDF membranes using the Trans-Blot Turbo system (Bio-Rad, USA). After blocking with skim milk, membranes were incubated overnight at 4 ℃ with primary antibodies against CD9, CD63, CD81, and VCAM-1 (all from Univ, China). Horseradish peroxidase (HRP)-conjugated secondary antibodies (Univ, China) were applied for 1 h at room temperature, and protein bands were visualized using a chemiluminescent imaging system (Bio-Rad ChemiDoc, USA).

### Cell selectivity and stability of TKEV

To optimize the hybrid ratio of TEV and KEV in TKEV, cellular uptake efficiency of different TKEV formulations was evaluated in 4T1 cells. Briefly, 4T1 cells were seeded in 24-well glass chamber slides at a density of 1 × 10⁵ cells per well and cultured overnight in complete medium. The medium was then replaced with various TKEV formulations and incubated for 4 h. After removal of the vesicle-containing medium, the cells were washed with PBS, fixed with 4% paraformaldehyde, and stained with DAPI. Cellular internalization of TKEV was visualized using confocal laser scanning microscopy (CLSM).

To assess the homologous targeting capability of TKEV, 4T1, HUVEC, HaCaT, and B16F10 cells were seeded on coverslips in 24-well plates and treated with DiO-labeled TKEV for 4 h. Cells were then fixed with paraformaldehyde, stained with DAPI, and imaged by CLSM.

The storage stability of KEV, TEV and TKEV was investigated by incubating the vesicles in PBS (pH 7.4) at 4 °C for 7 days. Particle size was monitored periodically by dynamic light scattering (DLS). To further assess their stability under physiological conditions, vesicles were also incubated in PBS containing 10% fetal bovine serum (FBS) at 37 °C for 24 h, followed by DLS measurement.

### Drug loading and stability of TKDS

DOX (Macklin, China) and siBcl2 (5′-GCAUGCGACCUCUGUUUGA-3′, Sangon Biotech, China) were electrostatically self-assembled into nanocomplexes (DS) as previously described [28]. Briefly, DOX and siBcl2 were mixed at various ratios and incubated briefly, after which siRNA condensation efficiency was assessed using agarose gel electrophoresis. TEV, KEV, or TKEV were then mixed with the DS complexes and co-extruded multiple times through polycarbonate membranes to facilitate vesicle reassembly and drug loading. Unencapsulated DS was removed by ultracentrifugation.

To investigate the release kinetics of DS from TKDS, suspensions of TKDS were loaded into dialysis bags (MWCO: 3500 Da, Millipore, Germany) and immersed in PBS at pH 7.4 or pH 5.5 under continuous shaking at 37 ℃. At predetermined time intervals, samples were withdrawn for drug quantification and replaced with fresh PBS to maintain sink conditions.

### Endocytosis and intracellular trafficking of TKDS

To investigate the cellular internalization pathways of TKDS, 4T1 cells were pretreated with chlorpromazine (10 µM), genistein (10 µM), or amiloride (10 µM) for 1 h, followed by incubation with DiO-labeled TKDS for 4 h. Cells were then harvested and analyzed by flow cytometry to quantify vesicle uptake.

For subcellular trafficking studies, 4T1 cells seeded on coverslips were incubated for 4 h with TKEV loaded with FAM-labeled siRNA and DOX (TKDS). After incubation, cells were fixed with paraformaldehyde, stained with DAPI, and imaged using confocal laser scanning microscopy (CLSM). To track temporal localization, TKDS was incubated with 4T1 cells for 0.5, 1, 2, and 4 h. Cells were then fixed, permeabilized with 0.1% Triton X-100, and blocked with 1% bovine serum albumin. Subsequently, samples were immunostained with primary antibodies against LAMP1 (Affinity, Norway), KDEL (Affinity, Norway), and Golgin-97 (Affinity, Norway) to label lysosomes, endoplasmic reticulum, and Golgi apparatus, respectively, followed by incubation with fluorescent secondary antibodies and CLSM imaging.

### In vitro gene expression and antitumor activity of TKDS

4T1 cells were seeded in 6-well plates and cultured for 24 h. Subsequently, the cells were treated with TKEV, DOX, siBcl2, or TKDS (all at a siRNA concentration of 20 nM). After 48 h of treatment, total RNA was extracted using TRIzol reagent (Thermo Fisher, USA). Then, 1 µg of RNA was reverse-transcribed into cDNA using a Fast RT Kit (Tiangen Biotech, China). Quantitative real-time PCR was performed on an ABI StepOne Plus Real-Time PCR System (USA) under the following cycling conditions: 40 cycles of 95 °C for 5 s and 60 °C for 40 s, using Talent qPCR PreMix (Tiangen Biotech, China).The sequences of the primers were as follows: Bcl2, forward: 5′-TGT GGC CTT CTT TGA GTT CG-3′; reverse: 5′-TAC CCA GCC TCC GTT ATC CT-3′; GAPDH, forward: 5′-GGT TGT CTC CTG CGA CTT CA-3; reverse: 5′-TGG TCC AGG GTT TCT TAC TCC-3′.

To evaluate the antitumor activity of TKDS, 4T1 cells were seeded in 96-well plates and treated with TKEV, DOX, siBcl2, or TKDS at various concentrations for 48 h. Cell viability was then assessed using the MTT assay. For apoptosis analysis, 4T1 cells were treated with different formulations at a siRNA concentration of 50 nM for 48 h. After treatment, cells were trypsinized, washed, and stained with an Annexin V-FITC apoptosis detection kit. Apoptotic rates were analyzed using a BD FACSVerse flow cytometer.

### Isolation and culture of immature DCs and T cells

Bone marrow cells were isolated from female BALB/c mice (8 weeks old) to generate dendritic cells (DCs). The cells were suspended in complete RPMI-1640 medium supplemented with 10% fetal bovine serum, 1% penicillin/streptomycin, 20 ng/mL GM-CSF, and 10 ng/mL IL-4 to induce DC differentiation. CD8⁺ T cells were isolated from spleens of BALB/c mice using an immunomagnetic bead-based kit (Dynabeads, Thermo Fisher Scientific).

### Measurement of DC-specific surface molecules

DCs treated with PBS (untreated), LPS, TEV, KEV, or TKEV were immunostained with a FITC-conjugated anti-CD11c antibody followed by APC-conjugated anti-CD80, PE-conjugated anti-CD86, and PE-Cy7–conjugated anti–MHC-II antibodies (all from BD Biosciences) for 30 min at 4 °C. After washing with PBS, the cells were analyzed using a flow cytometer.

### Measurement of T cell proliferation and activation

T cells were labeled with a CFSE Cell Division Tracking Kit (abcam) according to the manufacturer’s instructions. Untreated, LPS-, TEV-, KEV-, or TKEV-treated DCs were co-cultured with CFSE-labeled T cells. T cell proliferation was assessed by flow cytometry after staining with PerCP-conjugated anti-CD8 antibody (BD Biosciences), comparing results with non-proliferating control T cells. For T cell activation, co-cultures were immunostained with PE-Cy7–conjugated anti-CD107a and APC-conjugated anti–IFN-γ antibodies (BD Biosciences). IFN-γ levels in the supernatant were quantified using a commercial ELISA kit (BioLegend) following the manufacturer’s protocol.

### In vivo distribution of TKDS

Female BALB/c mice (6 weeks) were obtained from Shanghai JSJ Laboratory Animal Co., Ltd. All animal procedures were conducted in compliance with protocols approved by the Animal Research Committee of Shanghai Ocean University. A 4T1 cell suspension (1 × 10⁶ cells) was subcutaneously injected into the right flank of each mouse. When tumor volumes reached approximately 200 mm³, mice were intravenously injected with DiR-labeled KDS, TDS, or TKDS. At 6, 12, and 24 h post-injection, mice were euthanized, and major organs (heart, liver, spleen, lung, kidney) along with tumors were harvested for ex vivo fluorescence imaging using an IVIS Spectrum in vivo imaging system.

To evaluate the biodistribution of drug-loaded formulations, DS, KDS, TDS and TKDS were intravenously administered to BALB/c mice (*n* = 5 per group) at a DOX dose of 5 mg/kg. After 2 h, blood and tissue samples were collected, rinsed, homogenized, and DOX concentrations were quantified via HPLC.

### In vivo antitumor efficacy of TKDS

Subcutaneous xenografts were established by inoculating 4T1 cells (1 × 10⁶ cells) into the right flank of female BALB/c mice. When tumor volumes reached approximately 50 mm³, mice were randomly assigned to seven groups (*n* = 5): PBS, DS, KDS, TDS and TKDS. Treatments were administered intravenously every two days at doses of 100 µg DOX and 10 µg siBcl2 per injection. Tumor volumes and body weights were recorded throughout the study. After 14 days, all mice were euthanized, and major organs were collected for hematoxylin and eosin (H&E) staining.

### Statistical analysis

All quantitative data are expressed as mean ± standard deviation (SD) from at least three independent experiments. Differences between groups were assessed using Student’s t-test or one-way ANOVA where appropriate. A p-value < 0.05 was considered statistically significant (*p < 0.05, **p < 0.01, ***p < 0.001).

## Supplementary Information

Below is the link to the electronic supplementary material.


Supplementary Material 1.



Supplementary Material 2



Supplementary Material 3


## Data Availability

All data are available from the corresponding authors upon reasonable request.
