# Supplementary material for: Cross-species hybrid vesicles enable synergistic immunochemotherapy through enhanced targeted drug delivery and immune activation
Source: J Nanobiotechnology. 2026 Jun 19;24:820. doi: 10.1186/s12951-026-04699-2 (PMC13528136; doi:10.1186/s12951-026-04699-2)
Supplement: Supplementary file 1 [file 12951_2026_4699_MOESM1_ESM.docx]

Figure 1. Uncropped Blots images of CD9 (A), CD63 (B), CD81 (C) and VCAM-1 (D). (The images are presented without any cropping and clearly display the original protein band data across 3 lanes, including 2 marker lanes and 3 sample lanes.)

Figure 2. Original Western Blots of CD9.

Figure 3. Original Western Blots of CD63.

Figure 4. Original Western Blots of CD81.

Fig 5. Original Western Blots of VCAM-1.
