# Supplementary material for: Cross-species hybrid vesicles enable synergistic immunochemotherapy through enhanced targeted drug delivery and immune activation
Source: J Nanobiotechnology. 2026 Jun 19;24:820. doi: 10.1186/s12951-026-04699-2 (PMC13528136; doi:10.1186/s12951-026-04699-2)
Supplement: Supplementary file 2 [file 12951_2026_4699_MOESM2_ESM.docx]

Dear Prof. Samthangadaniel Vinipal Judu,

Thank you for your guidance regarding the technical check for our manuscript titled **“Cross-species hybrid vesicles enable synergistic immunochemotherapy through enhanced targeted drug delivery and immune activation”** (Manuscript ID: 6d769c65-eed5-447d-8621-0efb710667e1).

As requested, we have now uploaded a supplementary file containing the **full uncropped gels and blots images** for the proteins CD9, CD63, CD81, and VCAM-1. The images are presented without any cropping and clearly display the original protein band data across **3 lanes, including 2 marker lanes and 3 sample lanes**. We believe these unprocessed images retain the complete and reliable protein data relevant to the study.

All requested amendments have been made in the submission system, and no other changes have been introduced to the manuscript.

Please let us know if further information or adjustments are needed. We appreciate your support in moving the submission forward.

Yours sincerely,

Kehai Liu

PhD, Professor

Shanghai Ocean University
