# Supplementary material for: Cross-species hybrid vesicles enable synergistic immunochemotherapy through enhanced targeted drug delivery and immune activation
Source: J Nanobiotechnology. 2026 Jun 19;24:820. doi: 10.1186/s12951-026-04699-2 (PMC13528136; doi:10.1186/s12951-026-04699-2)
Supplement: Supplementary file 3 [file 12951_2026_4699_MOESM3_ESM.docx]

**Supplementary information**

**Cross-species hybrid vesicles enable synergistic immunochemotherapy through enhanced targeted drug delivery and immune activation**

**Zhou Fang ^a^, Saichao Wei ^a^, Jiaqi Xu ^a^, Min Zhang ^a^, Mingyu Yin ^a^, Jun Wang ^b, *^, Kehai Liu ^a, *^**

^a^ *Department of Biopharmaceutical Science, Shanghai Ocean University, Hucheng Ring Road, Shanghai, China*

^b^ *The International Peace Maternal and Child Health Hospital, School of Medicine, Shanghai Jiao Tong University, Shanghai, China*

**Contents**

Supplementary Figure S1. The concentration of TGF-β in fresh TEV, TEV subjected to multiple freeze-thaw cycles, and TKEV was measured by ELISA.

Supplementary Figure S2. 4T1 cell uptake of TKEV in different membrane fusion formulations was evaluated by CLSM.

Supplementary Figure S3. TKEV prepared with various formulations were incubated in PBS containing 10% FBS at 37°C for 0–4 hours.

Supplementary Figure S4. Full uncropped gels of CD9, CD63, CD81 and VCAM-1.

Supplementary Figure S5. TEV and KEV were labeled with DiO and DiD, respectively, and fused at ratios of 1:0, 1:1, 1:3, 1:7, and 0:1.

Supplementary Figure S6. Stability of KEV, TEV and TKEV over the period time in terms of size change at 4℃.

Supplementary Figure S7. Intracellular trafficking analysis of TKDS.

Supplementary Figure S8. Representation flow cytometry plots of mature DCs (CD80, CD86 and MHC-II) after a 24 h treatment with PBS, LPS, TEV, KEV and TKEV.

Supplementary Figure S9. Representation flow cytometry plots of CD8^+^CD107a^+^ and CD8^+^IFN-γ^+^ T cell.

Supplementary Figure S10. KDS, TDS, and TKDS were incubated in PBS containing 10% FBS at 37°C for 0–4 hours.

Supplementary Figure S11. Representative CLSM images of different groups absorbed in the tumor. Scale bar = 50 μm.

Supplementary Figure S12. Body weight changes of mice during the treatment period (n = 5).

Supplementary Figure S13. Representative flow cytometry plots and quantitative analysis of mature DCs in TDLN.

Supplementary Figure S14. Representative flow cytometry plots and quantitative analysis of activated CD8^+^ T cells in TDLN.

Supplementary Figure S15. Representative flow cytometry plots and quantitative analysis of CD8^+^ T cells in the tumor microenvironment.

Supplementary Figure S16. Representative flow cytometry plots and quantitative analysis of CD8^+^IFN-γ^+^ T cells in the tumor microenvironment.

Supplementary Figure S17. H&E staining of major organs. Scale bar = 50 μm.

**Supplementary Figure S1.** The concentration of TGF-β in fresh TEV, TEV subjected to multiple freeze-thaw cycles, and TKEV was measured by ELISA. Data are presented as mean ± SD (n = 3). ****p* < 0.001.

**Supplementary Figure S2.** 4T1 cell uptake of TKEV in different membrane fusion formulations was evaluated by CLSM. scale bar = 20 μm

**Supplementary Figure S3.** TKEV prepared with various formulations were incubated in PBS containing 10% FBS at 37°C for 0–4 hours. Particle size was monitored at the indicated time points by dynamic light scattering (DLS). Data are presented as mean ± SD (n = 3). **p* < 0.05; ***p* < 0.01; ****p* < 0.001.

**Supplementary Figure S4**. Full uncropped gels of CD9, CD63, CD81 and VCAM-1.

**Supplementary Figure S5.** TEV and KEV were labeled with DiO and DiD, respectively, and fused at ratios of 1:0, 1:1, 1:3, 1:7, and 0:1. The fluorescence intensity of hybrid vesicles was analyzed by flow cytometry. Data are presented as mean ± SD (n = 3).

**Supplementary Figure S6**. Stability of KEV, TEV and TKEV over the period time in terms of size change at 4℃ ((n = 5).

**Supplementary Figure S7**. Intracellular trafficking analysis of TKDS.

**Supplementary Figure S8**. Representation flow cytometry plots of mature DCs (CD80, CD86 and MHC-II) after a 24 h treatment with PBS, LPS, TEV, KEV and TKEV.

**Supplementary Figure S9.** Representation flow cytometry plots of CD8^+^CD107a^+^ and CD8^+^IFN-γ^+^ T cell.

**Supplementary Figure S10.** Representative CLSM images of different groups absorbed in the tumor. Scale bar = 50 μm

**Supplementary Figure S11.** KDS, TDS, and TKDS were incubated in PBS containing 10% FBS at 37°C for 0–4 hours. Particle size was monitored at the indicated time points by dynamic light scattering (DLS). Data are presented as mean ± SD (n = 3). ****p* < 0.001.

**Supplementary Figure S12.** Body weight changes of mice during the treatment period (n = 5).

**Supplementary Figure S13.** Representative flow cytometry plots and quantitative analysis of mature DCs in TDLN.

**Supplementary Figure S14.** Representative flow cytometry plots and quantitative analysis of activated CD8^+^ T cells in TDLN.

**Supplementary Figure S15.** Representative flow cytometry plots and quantitative analysis of CD8^+^ T cells in the tumor microenvironment.

**Supplementary Figure S16.** Representative flow cytometry plots and quantitative analysis of CD8^+^IFN-γ^+^ T cells in the tumor microenvironment.

**Supplementary Figure S17.** H&E staining of major organs. Scale bar = 50 μm.
